# Communication and informed consent should be counted as quality failures in childbirth care (letter to the editors)

**DOI:** 10.1016/j.xagr.2026.100631

**Published:** 2026-03-26

**Authors:** Aaron A. Funa

**Affiliations:** College of Health and Science, Sorsogon State University, Sorsogon City, Philippines; Graduate School, Sorsogon State University, Sorsogon City, Philippines

To the Editors:

I read with interest the article by Aloui et al^1^ on mistreatment during childbirth in a Tunisian maternity unit. The authors’ finding that many women experienced verbal abuse, poor communication, lack of information, and failures of consent is important because it shows that disrespect in childbirth is not limited to dramatic or exceptional events. It can also occur through routine clinical interactions that are normalized in maternity settings.

Of note, 1 point deserves further emphasis. Failures of communication and informed consent in childbirth should not be treated merely as interpersonal or ethical concerns that exist alongside technically competent care. Women not being adequately informed, women being excluded from decision-making, or women undergoing procedures without meaningful consent are variables that should be considered as quality failures in care delivery. Framing them only as matters of professionalism minimizes their clinical and institutional importance.

This distinction is important because women’s experiences are linked to maternal and newborn outcomes. In the *American Journal of Obstetrics & Gynecology Global Reports*, Sudhinaraset et al^2^ showed that better person-centered maternity care was associated with lower risks of maternal complications, newborn complications, and postpartum depression. Together with Aloui et al,^1^ these findings suggest that respectful interaction is not separate from safe care. It is part of what makes care effective, acceptable, and responsive.

Therefore, routine maternity care audits should not only move beyond counting only clinical adverse events but also include patient-reported measures of information giving, consent, respectful communication, and involvement in decision-making. Unless these domains are measured, they remain easy to ignore. Aloui et al^1^ have already demonstrated the scale of the problem. The next step is to define these domains as measurable indicators of obstetrical quality rather than as optional features of compassionate care.

## CRediT authorship contribution statement

**Aaron A. Funa:** Conceptualization, Formal analysis, Investigation, Writing – original draft, Writing – review & editing.
